# The Correlation Between Bladder Cancer and Obesity, Overweight, Physical Inactivity, and Tobacco Use: An Ecological Study in Asian Countries

**DOI:** 10.5334/aogh.2545

**Published:** 2019-07-10

**Authors:** Fatemeh Rezaei, Hamid-Reza Tabatabaee, Vahid Rahmanian, Alireza Mirahmadizadeh, Soheil Hassanipour

**Affiliations:** 1 Shiraz University of Medical Sciences, Shiraz, IR; 2 Research Center for Health Sciences, Institute of Health, Department of Epidemiology, School of Health, Shiraz University of Medical Sciences, Shiraz, IR; 3 Research Center for Social Determinant of Health, Jahrom University of Medical Sciences, Jahrom, IR; 4 Non-communicable Diseases Research Center, Shiraz University of Medical Sciences, Shiraz, IR; 5 Gastrointestinal and Liver Diseases Research Center, Guilan University of Medical Sciences, Rasht, IR

## Abstract

**Background:**

Bladder cancer is the ninth most common cancer in the world.

**Objectives:**

This study aimed to determine the correlation between age-standardized incidence rates of bladder cancer and some risk factors in Asian countries through an extensive ecological analysis.

**Methods:**

This ecological study evaluated the correlation between age-standardized incidence rates of bladder cancer and obesity, overweight, physical inactivity, and tobacco use in 30 Asian countries. To determine the factors that were significantly related to age-standardized incidence rate of bladder cancer, a univariate analysis was performed using simple linear regression. In the next step, variables with p-values less than 0.25 were entered into a multivariate linear regression model.

**Results:**

The incidence of bladder cancer was higher in countries with higher prevalence of overweight (r^2^ = 0.36, p < 0.001), obesity (r^2^ = 0.34, p = 0.001), current daily tobacco use (r^2^ = 0.17, p = 0.03), and physical inactivity (r^2^ = 0.13, p = 0.04). The results of multiple regression analysis indicated a direct correlation between the incidence of bladder cancer and overweight (β = 0.15, p < 0.001) and current daily tobacco use (β = 0.21, p = 0.001).

**Conclusions:**

There was a significant relationship between the incidence of bladder cancer and overweight and current daily tobacco use. Further epidemiological studies are needed to confirm this relationship.

## Introduction

Bladder cancer is the ninth most common cancer in the world, with approximately more than 450,000 new cases in 2015 [1]. More than 60% of bladder cancer cases and half of the 165,000 related mortality occur in less-developed countries [2,3]. Bladder cancer survival is also lower in developing and less-developed countries [4,5]. Risk factors for bladder cancer include age, sex, smoking, exposure to arsenic in drinking water, occupational exposure to aromatic amines, schistosomiasis, some medications such as phenacetin-containing analgesics, and family history of bladder cancer [6,7,8,9,10,11,12]. However, there is lack of evidence about the relationship between the risk of bladder cancer and consumption of Egyptian water-pipe [7]. Additionally, obesity seems to elevate the risk of bladder cancer [13]. On the other hand, physical activity may decrease the risk of bladder cancer because it indirectly reduces obesity and helps maintain body weight [14]. Considering the fact that many of these risk factors can be improved through lifestyle modifications and environmental protection interventions, several steps should be taken to prevent cancer. In order to succeed in preventive interventions, the role of risk factors must first be identified. Many studies that have examined the relationship between smoking and bladder cancer have demonstrated that cigarette smoking increased the risk of this cancer [15,16,17]. In the present study, the association between tobacco use (both cigarette and hookah) and bladder cancer was investigated. Contrary to the fact that there is general agreement on the relationship between risk factors such as cigarettes smoking and bladder cancer, there is no clear agreement on some other risk factors such as obesity and physical activity [14,18,19,20,21,22,23,24]. Epidemiological studies play an important role in this field. In this context, a wide range of data related to obesity, overweight, physical activity, and tobacco use is available internationally and in various databases that would be a potential advantage for ecological studies. Therefore, the present study researchers, with awareness of the problems of ecological studies, conducted this study to make hypotheses in a large population. This study aimed to determine the correlation between bladder cancer and four risk factors; i.e., obesity, overweight, physical inactivity, and tobacco use, in Asian countries.

## Materials and Methods

This was an ecological study in which researchers assessed aggregate variables. In order to collect the variables, a dataset was prepared containing information from each country based on the age-standardized incidence rate of bladder cancer and the prevalence of obesity, overweight, smoking, and physical inactivity. In total, the study included 30 Asian countries with complete data for each of the four above-mentioned variables. The age-standardized incidence rates of bladder cancer in Asian countries were derived from the website of the International Agency for Research on Cancer (IARC) GLOBOCAN database in 2012 (the last year when complete data were available). It should be noted that GLOBOCAN uses national registries and vital records to estimate the annual age-standardized incidence rates of bladder cancer per 100,000 population. Information on the prevalence of obesity, overweight, physical inactivity, and current daily tobacco use was obtained from the Non-Communicable Diseases (NCDs) country profiles of 2011. Researchers gathered this information was from a variety of sources that provided relevant data, including countries, World Health Organization (WHO) estimations, and results of a global review of the assessment of national capacity for NCDs prevention and control conducted in 2009/2010 [25]. In case the information was not mentioned in the references, other sources of statistics were considered. For instance, physical inactivity data for Bahrain, Jordan, Oman, Qatar, Syria, and Uzbekistan were derived from a study on the prevalence of physical inactivity in Muslim countries [26]. The data for Kyrgyzstan were also obtained from a study on the prevalence of risk factors for noninvasive diseases [27]. Additionally, the obesity and overweight data for Georgia were not mentioned in the reference and, consequently, the required information was obtained from ‘Overweight and Obesity in Georgia’ in 2005 [28]. Moreover, data on the prevalence of tobacco use in some countries were not mentioned in the references and there was no information available from other sources. Hence, the incidence of tobacco use in Bhutan was collected from ‘WHO Report on the Global Tobacco Epidemic 2013’ and data for Qatar and Kazakhstan were gathered from ‘WHO Country Profiles on the Tobacco Epidemic.’ The results of a survey were also used to obtain the incidence of daily smoking in Syria. Accordingly, 29%, 1%, and 1% of the subjects used cigarettes, hookahs, and both, respectively [29].

In the present study, current daily tobacco use was defined as the percentage of individuals aged 15 years and over who used tobacco every day. Additionally, physical inactivity was regarded as the percentage of individuals aged 15 years and over who had moderate physical activity for less than 30 minutes a week or intense physical activity for less than 20 minutes three times a week. Finally, the percentage of individuals aged 20 years and over with Body Mass Index (BMI) = 30 kg/m^2^ and BMI ≥25 kg/m^2^ was considered as obesity and overweight, respectively [25].

### Statistical analysis

Scatter plots were drawn for the age-standardized incidence rate of bladder cancer in all countries based on smoking, obesity, overweight, and physical inactivity. Univariate linear regression was used to determine the association between the age-standardized incidence rate of bladder cancer and the study variables. In the next stage, variables with p-values less than 0.25 in the univariate linear regression were entered into the multivariate linear regression model.

## Results

**Figure 1:**
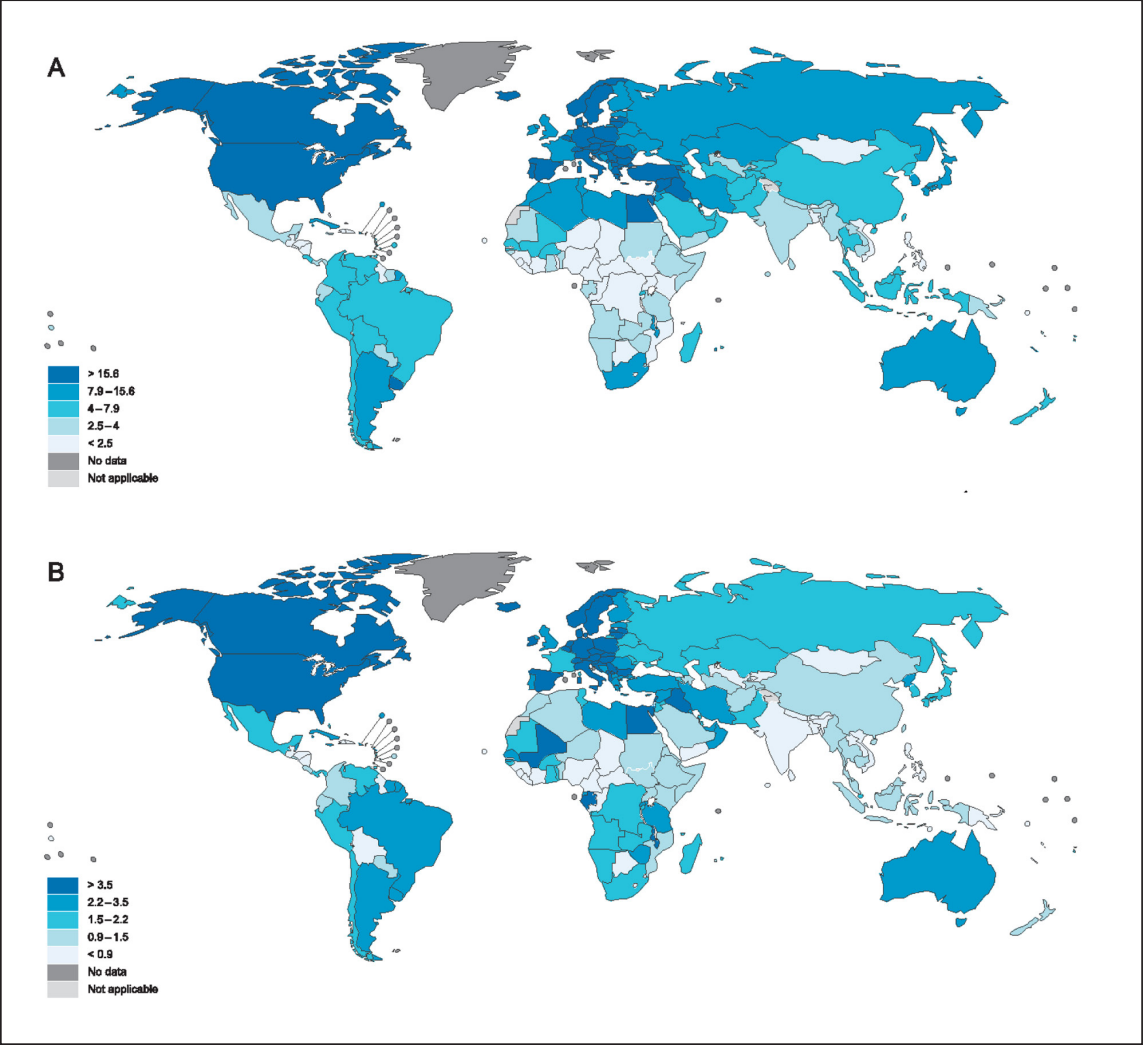
Estimated age-standardised incidence rates of bladder cancer in 2012 in **(A)** men and **(B)** women.

The estimated age-standardised incidence rates of bladder cancer in 2012 was presented in Figure 1 [3]. The highest incidence of bladder cancer in Asian countries was 16.60 per 100,000 in Lebanon, while the lowest was 1.10 per 100,000 in Vietnam. In addition, the highest incidence of current daily tobacco use was 36.8% in Lebanon and the lowest was 4% in Oman. Besides, the highest prevalence of physical inactivity was 67.8% in Bahrain, while the lowest was 4.7% in Bangladesh. The highest prevalence of obesity was 42% in Kuwait and the lowest was 1.1% in Bangladesh. Also, the highest prevalence of overweight was 78.8% in Kuwait and the lowest was 7.6% in Bangladesh.

The univariate linear regression model for smoking, physical inactivity, obesity, and overweight has been presented in Table 1. The results showed a significant correlation between the incidence of bladder cancer and current daily tobacco use (R^2^ = 0.17), physical inactivity (R^2^ = 0.13), obesity (R^2^ = 0.34), and overweight (R^2^ = 0.36). In the next step, multivariate linear regression with stepwise method was used to determine the variables correlated to bladder cancer. In so doing, the variables with p-values less than 0.25 in the univariate analysis were entered into the model. The results revealed a significant correlation between the incidence of bladder cancer and overweight (β = 0.15, p < 0.001) and current daily tobacco use (β = 0.21, p = 0.001) (Table 2).

**Table 1:** The factors related to the age-standardized incidence rate of bladder cancer in the univariate model.

Variables	Regression coefficient (*B*)	SE	T	*P*-value	95% CI for *B*

**Current daily tobacco use**	0.16	0.07	0.63	0.03	0.009–0.32
**Physical inactivity**	0.06	0.03	2.06	0.04	0.00–0.1
**Obesity**	0.15	0.04	3.86	0.001	0.07–0.23
**Overweight**	0.08	0.02	4.09	<0.001	0.04–0.13

SE: Standard Error; CI: Confidence Interval.

**Table 2:** The factors significantly related to the incidence rate of bladder cancer in multivariate linear regression model.

Model	R	R^2^	R^2^ adjusted	F	*P*-value	B	T	*P*-value

**Overweight**	0.77	0.59	0.56	19.92	<0.001	0.15	5.49	<0.001
**Current daily tobacco use**						0.21	3.85	0.001

**Figure 2:**
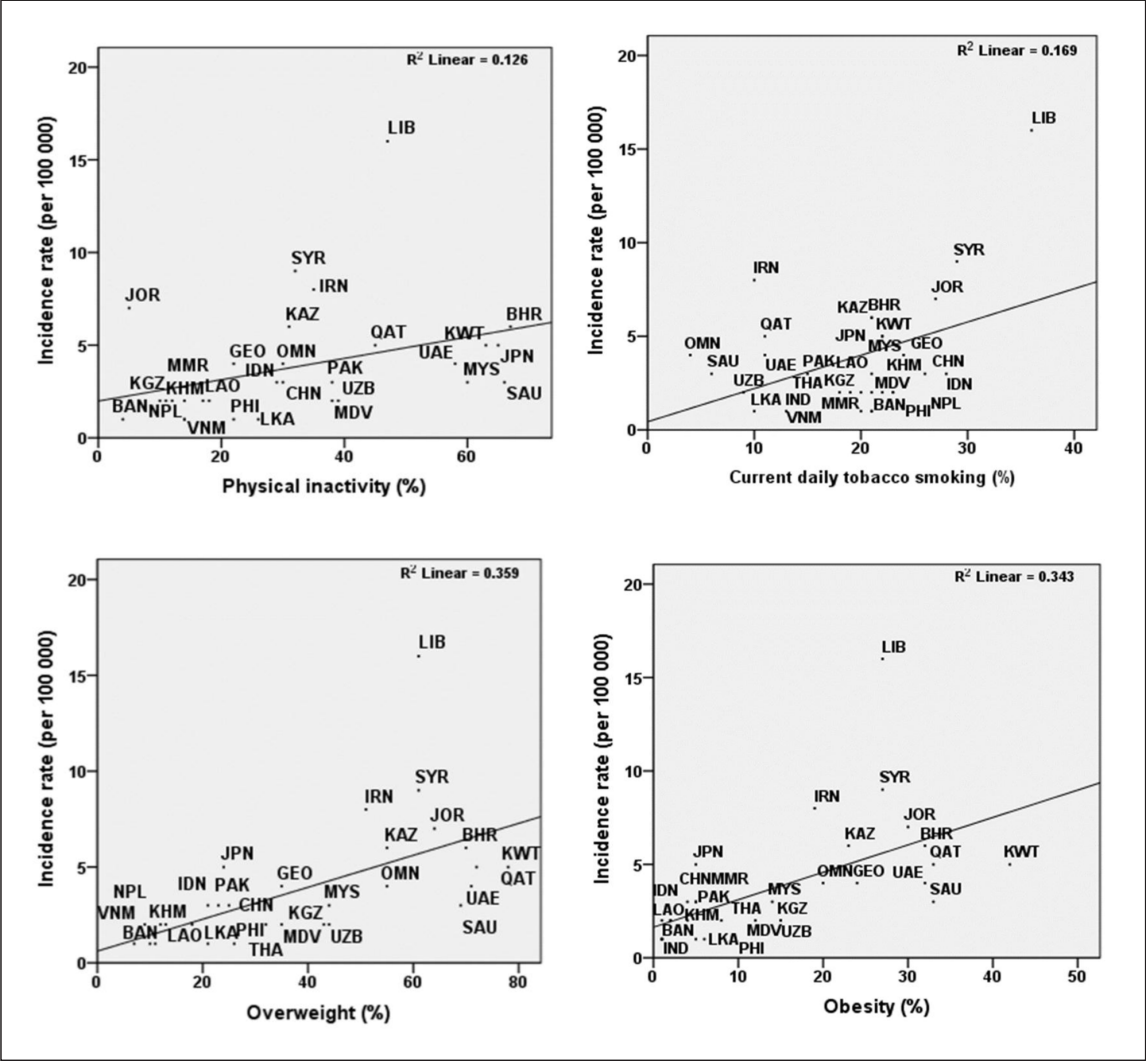
Age-standardized incidence rates of bladder cancer based on current daily tobacco use, physical inactivity, overweight, and obesity in 30 countries. BHR, Bahrain; BAN, Bangladesh; KHM, Cambodia; CHN, China; GEO, Georgia; IND, India; IDN, Indonesia; IRN, Iran; JPN, Japan; JOR, Jordan; KAZ, Kazakhstan; KWT, Kuwait; KGZ, Kyrgyzstan; LAO, Lao PDR; LIB, Lebanon; MYS, Malaysia; MDV, Maldives; MMR, Myanmar; NPL, Nepal; OMN, Oman; PAK, Pakistan; PHI, Philippines; QAT, Qatar; SAU, Saudi Arabia; LKA, Sri Lanka; SYR, Syria; THA, Thailand.

The scatter plot of the standardized incidence rate of bladder cancer in terms of daily tobacco use, physical inactivity, obesity, and overweight has been depicted in Figure 2. Accordingly, the highest R^2^ was reported for overweight followed by obesity. The collinearity between the variables was also evaluated in this study. The results demonstrated no collinearity between obesity and physical inactivity as well as between obesity and overweight (VIF = 1).

## Discussion

The results of this study revealed that the age-standardized incidence rate of bladder cancer was directly correlated to overweight and current daily tobacco use. Until now, some studies have been conducted on other cancers [30,31,32,33,34,35,36]. However, the authors could not find any ecological studies on the effects of smoking, physical inactivity, obesity, and overweight on bladder cancer. The present study indicated a significant association between bladder cancer and being overweight, but not between obesity and bladder cancer. A prior study demonstrated that obesity was associated with an increased risk of bladder cancer and particularly its progression, recurrence, and mortality [37]. According to the Institute for Health Metrics and Evaluation, 37% of the world’s population was overweight or obese. It was also reported that the prevalence of obesity and overweight has increased by 27.5% in adults and by 47.1% in children since 1980 [38]. Although the present study findings revealed no significant relationships between obesity and bladder cancer, several meta-analytic results have demonstrated that higher BMI was associated with an elevation in endometrial, colorectal, and postmenopausal breast cancer [39,40,41]. Increased BMI was also related to a higher risk of esophageal adenocarcinoma, thyroid cancer, renal cancer, multiple myeloma, gallbladder cancer, leukemia, pancreatic cancer, non-Hodgkin lymphoma, and ovarian cancer [39,42,43]. Moreover, obesity has been reported to be associated with other noninvasive diseases, such as glucose intolerance, insulin resistance, hyperinsulinemia, and type II diabetes [44,45]. Given the fact that obesity and overweight are following an ascending trend in the world and considering the findings of the present study, further interventions are necessary to control or reduce the rate of overweight and obesity worldwide. Although understanding the link between obesity and cancer can be helpful in treatment of this disease, prevention of overweight and obesity remains to be one of the priorities [46].

The current study findings revealed no significant relationships between bladder cancer and physical inactivity. Similar results were also obtained in other studies [37,47]. However, some other studies showed that physical inactivity was positively related to bladder cancer [48,49]. Indeed, higher levels of physical activity were associated with lower risks of breast cancer, colon cancer, diabetes, ischemic heart disease, and ischemic stroke [50]. The results of a meta-analysis also indicated that higher levels of physical leisure activities were related to lower risks of esophageal adenocarcinoma and liver, lung, kidney, gastric cardia, endometrial, myeloid leukemia, myeloma, colon, head and neck, rectal, and breast cancers [49]. The non-significant relationship observed in the present study might be attributed to the confounders whose information was not available. Therefore, supporting the promotion of physical activity is an important component in the prevention and control of many cancers and other NCDs.

The results of the present study demonstrated a significant relationship between bladder cancer and current daily tobacco use, which is consistent with the results of other studies conducted on the issue [15,16,17,51]. Smoking tobacco has been reported to be a strong risk factor for bladder cancer with Population Attributable Risk (PARs) of approximately 50% in both males and females [15]. It has also been shown that the risk of bladder cancer was 2–6 times higher in smokers than in non-smokers [52]. Moreover, it has been estimated that smoking caused about 31% deaths due to bladder cancer in males and 14% in females worldwide [53]. In addition to bladder cancer, smoking has been reported to be associated with pancreatic, lung, stomach, breast, colon, and rectal cancers [54,55,56,57,58,59,60]. Additionally, smoking increased the risk of mortality of lung, larynx, bile duct, esophagus, liver, stomach, and pancreas cancers and leukemia [51]. Since smoking is an independent risk factor for many cancers as well as other NCDs, control programs for smoking are essential to reduce the epidemic of cigarette-related illnesses.

### Limitation

As mentioned above, aggregate data were used in this study. Therefore, the findings might have not resulted from the probability of ecological fallacy applied to individuals. This is one of the limitations of ecological studies. For instance, all individuals living in areas with a high incidence of overweight or smoking may not be at risk of bladder cancer. Since ecological studies are more suitable for making hypotheses to provide definitive results, accurate case studies or cohorts are required to confirm the hypotheses presented in this investigation. Another limitation of the present study was that the prevalence of smoking and physical inactivity was evaluated in the ≥15 years age group and the prevalence of obesity and overweight was assessed among the individuals aged 20 years and above. Thus, inequalities of age groups regarding these variables may have affected their relationships with bladder cancer.

## Conclusion

There was a significant relationship between the incidence of bladder cancer and overweight and current daily tobacco use. Further epidemiological studies are needed to confirm this relationship.

## References

[1] Bray F, Ferlay J, Soerjomataram I, Siegel RL, Torre LA and Jemal A. Global cancer statistics 2018: GLOBOCAN estimates of incidence and mortality worldwide for 36 cancers in 185 countries. CA: A Cancer Journal for Clinicians. 2018; 68(6): 394–424. DOI: 10.3322/caac.21492 30207593

[2] Wong MCS, Fung FDH, Leung C, Cheung WWL, Goggins WB and Ng CF. The global epidemiology of bladder cancer: A joinpoint regression analysis of its incidence and mortality trends and projection. Scientific Reports. 2018; 8(1): 1129–1129. DOI: 10.1038/s41598-018-19199-z 29348548 PMC5773684

[3] Antoni S, Ferlay J, Soerjomataram I, Znaor A, Jemal A and Bray F. Bladder cancer incidence and mortality: A global overview and recent trends. European Urology. 2017; 71(1): 96–108. DOI: 10.1016/j.eururo.2016.06.010 27370177

[4] Pakzad R, Mohammadian-Hafshejani A, Mohammadian M, et al. Incidence and mortality of bladder cancer and their relationship with development in Asia. Asian Pacific Journal of Cancer Prevention. 2015; 16(16): 7365–7374. DOI: 10.7314/APJCP.2015.16.16.7365 26514538

[5] Hassanipour S, Delam H, Fathalipour M, Sharifi M, et al. The incidence of bladder cancer in Iran: a systematic review and meta-analysis. World Cancer Research Journal. 2019; 6(e1222): 1–10. DOI: 10.18502/ijpho.v9i3.1170

[6] Bachir BG and Kassouf W. Cause–effect? Understanding the risk factors associated with bladder cancer. Expert Review of Anticancer Therapy. 2012; 12(12): 1499–1502. DOI: 10.1586/era.12.140 23253215

[7] Kuper H, Boffetta P and Adami HO. Tobacco use and cancer causation: Association by tumour type. Journal of Internal Medicine. 2002; 252(3): 206–224. DOI: 10.1046/j.1365-2796.2002.01022.x 12270001

[8] Stewart SL, Cardinez CJ, Richardson LC, et al. Surveillance for cancers associated with tobacco use—United States, 1999–2004, 2008 Department of Health & Human Services, Center for Disease Control and Prevention. 18772853

[9] Volanis D, Kadiyska T, Galanis A, Delakas D, Logotheti S and Zoumpourlis V. Environmental factors and genetic susceptibility promote urinary bladder cancer. Toxicology Letters. 2012; 193(2): 131–137. DOI: 10.1016/j.toxlet.2009.12.018 20051252

[10] Pelucchi C, Bosetti C, Negri E, Malvezzi M and La Vecchia C. Mechanisms of disease: The epidemiology of bladder cancer. Nature Reviews Urology. 2006; 3(6): 327 DOI: 10.1038/ncpuro0510 16763645

[11] Fernandez MI, Brausi M, Clark PE, et al. Epidemiology, prevention, screening, diagnosis, and evaluation: Update of the ICUD-SIU joint consultation on bladder cancer. World Journal of Urology. 2019; 37(1): 3–13. DOI: 10.1007/s00345-018-2436-y 30105454

[12] Hashim D and Boffetta P. Occupational and environmental exposures and cancers in developing countries. Annals of Global Health. 2014; 80(5): 393–411. DOI: 10.1016/j.aogh.2014.10.002 25512155

[13] Qin Q, Xu X, Wang X and Zheng X-Y. Obesity and risk of bladder cancer: A meta-analysis of cohort studies. Asian Pacific Journal of Cancer Prevention. 2013; 14(5): 3117–3121. DOI: 10.7314/APJCP.2013.14.5.3117 23803089

[14] Koebnick C, Michaud D, Moore SC, et al. Body mass index, physical activity, and bladder cancer in a large prospective study. Cancer Epidemiology and Prevention Biomarkers. 2008; 17(5): 1214–1221. DOI: 10.1158/1055-9965.EPI-08-0026 18483344

[15] Freedman ND, Silverman DT, Hollenbeck AR, et al. Association between smoking and risk of bladder cancer among men and women. Jama. 2011; 306(7): 737–745. DOI: 10.1001/jama.2011.1142 21846855 PMC3441175

[16] Cumberbatch MG, Rota M, Catto JW and La Vecchia C. The role of tobacco smoke in bladder and kidney carcinogenesis: A comparison of exposures and meta-analysis of incidence and mortality risks. European Urology. 2016; 70(3): 458–466. DOI: 10.1016/j.eururo.2015.06.042 26149669

[17] Antoni S, Ferlay J, Soerjomataram I, Znaor A, Jemal A and Bray F. Bladder cancer incidence and mortality: A global overview and recent trends. European Urology. 2017; 71(1): 96–108. DOI: 10.1016/j.eururo.2016.06.010 27370177

[18] Attner B, Landin-Olsson M, Lithman T, Noreen D and Olsson H. Cancer among patients with diabetes, obesity and abnormal blood lipids: A population-based register study in Sweden. Cancer Causes & Control. 2012; 23(5): 769–777. DOI: 10.1007/s10552-012-9946-5 22467266

[19] Holick CN, Giovannucci EL, Stampfer MJ and Michaud DS. Prospective study of body mass index, height, physical activity and incidence of bladder cancer in US men and women. International Journal of Cancer. 2007; 120(1): 140–146. DOI: 10.1002/ijc.22142 17036323

[20] Møller H, Mellemgaard A, Lindvig K and Olsen JH. Obesity and cancer risk: A Danish record-linkage study. European Journal of Cancer. 1994; 30(3): 344–350. DOI: 10.1016/0959-8049(94)90254-2 8204357

[21] Tripathi A, Folsom AR and Anderson KE. Risk factors for urinary bladder carcinoma in postmenopausal women. Cancer. 2002; 95(11): 2316–2323. DOI: 10.1002/cncr.10975 12436437

[22] Wannamethee S, Shaper A and Walker M. Physical activity and risk of cancer in middle-aged men. British Journal of Cancer. 2001; 85(9): 1311 DOI: 10.1054/bjoc.2001.2096 11720466 PMC2375260

[23] Wolk A, Gridley G, Svensson M, et al. A prospective study of obesity and cancer risk (Sweden). Cancer Causes & Control. 2001; 12(1): 13–21. DOI: 10.1023/A:1008995217664 11227921

[24] Lee YC and Hashibe M. Tobacco, alcohol, and cancer in low- and high-income countries. Annals of Global Health. 2014; 80(5): 378–383. DOI: 10.1016/j.aogh.2014.09.010 25512153

[25] Ozlen F, Kafadar AM, Abuzayed B, et al. Surgical treatment of trigonocephaly: Technique and long-term results in 48 cases. Journal of Neurosurgery Pediatrics. 2001; 7(3): 300–310. DOI: 10.3171/2010.12.PEDS10359 21361772

[26] Kahan D. Adult physical inactivity prevalence in the Muslim world: Analysis of 38 countries. Preventive Medicine Reports. 2015; 2: 71–75. DOI: 10.1016/j.pmedr.2014.12.007 26844051 PMC4721436

[27] Makhmutkhodzhaev S, Kydyralieva R, Altymysheva A, et al. Prevalence of risk factors of non-communicable disease in Kyrgyzstan: Assessment using WHO STEPS approach. Kardiologiia. 2001; 56(11): 86–90. DOI: 10.18565/cardio.2016.11.86-90 28290823

[28] Mircevsk V, Zogovska E, Chaparoski A, et al. Trigonocephaly – Our experience and treatment in the republic of Macedonia. Prilozi (Makedonska akademija na naukite i umetnostite Oddelenie za medicinski nauki). 2017; 38(1): 35–40. DOI: 10.1515/prilozi-2017-0004 28593893

[29] Ward K, Eissenberg T, Rastam S, et al. The tobacco epidemic in Syria. Tobacco Control. 2015; 15(suppl 1): i24–i29. DOI: 10.1136/tc.2005.014860 PMC2563543 16723671

[30] Grant WB. An ecologic study of dietary and solar ultraviolet-B links to breast carcinoma mortality rates. Cancer. 2002; 94(1): 272–281. DOI: 10.1002/cncr.10196 11815987

[31] Grant WB. A multicountry ecologic study of risk and risk reduction factors for prostate cancer mortality. European Urology. 2004; 45(3): 271–279. DOI: 10.1016/j.eururo.2003.08.018 15036670

[32] Sasaki S, Horacsek M and Kesteloot H. An ecological study of the relationship between dietary fat intake and breast cancer mortality. Preventive Medicine. 1993; 22(2): 187–202. DOI: 10.1006/pmed.1993.1016 8483858

[33] Wynder EL, Taioli E and Fujita Y. Ecologic study of lung cancer risk factors in the US and Japan, with special reference to smoking and diet. Japanese Journal of Cancer Research. 1992; 83(5): 418–423. DOI: 10.1111/j.1349-7006.1992.tb01944.x 1618693 PMC5918856

[34] Hassanipour-Azgomi S, Mohammadian-Hafshejani A, Ghoncheh M, Towhidi F, Jamehshorani S and Salehiniya H. Incidence and mortality of prostate cancer and their relationship with the Human Development Index worldwide. Prostate Int. 2016; 4(3): 118–124. DOI: 10.1016/j.prnil.2016.07.001 27689070 PMC5031898

[35] Hassanipour S, Mohammadian-Hafshejani A, Ghoncheh M and Salehiniya H. The incidence and mortality of esophageal cancer and its relationship with development in the world. Biomedical Research and Therapy. 2017; 4(9): 1607–1623. DOI: 10.15419/bmrat.v4i9.368

[36] Salehiniya H, Pakzad R, Hassanipour S and Mohammadian M. The incidence and mortality of thyroid cancer and its relationship with HDI in the world. World Cancer Research Journal. 2018; 5(2): 1–6.

[37] Noguchi JL, Liss MA and Parsons JK. Obesity, physical activity and bladder cancer. Current Urology Reports. 2015; 16(10): 74 DOI: 10.1007/s11934-015-0546-2 26303776

[38] Mahdavifar N, Pakzad R, Ghoncheh M, Gandomani H and Salehiniya H. Epidemiology, incidence, and mortality of gallbladder cancer and its relation with development in the world. Annals of Tropical Medicine and Public Health. 2017; 10(3): 563–570. DOI: 10.7603/s40730-016-0048-y

[39] Reeves GK, Pirie K, Beral V, Green J, Spencer E and Bull D. Cancer incidence and mortality in relation to body mass index in the Million Women Study: Cohort study. Bmj. 2007; 335(7630): 1134 DOI: 10.1136/bmj.39367.495995.AE 17986716 PMC2099519

[40] Larsson SC and Wolk A. Obesity and colon and rectal cancer risk: A meta-analysis of prospective studies. The American Journal of Clinical Nutrition. 2007; 86(3): 556–565. DOI: 10.1093/ajcn/86.3.556 17823417

[41] Moghaddam AA, Woodward M and Huxley R. Obesity and risk of colorectal cancer: A meta-analysis of 31 studies with 70,000 events. Cancer Epidemiology and Prevention Biomarkers. 2007; 16(12): 2533–2547. DOI: 10.1158/1055-9965.EPI-07-0708 18086756

[42] Renehan AG, Tyson M, Egger M, Heller RF and Zwahlen M. Body-mass index and incidence of cancer: A systematic review and meta-analysis of prospective observational studies. The Lancet. 2008; 371(9612): 569–578. DOI: 10.1016/S0140-6736(08)60269-X 18280327

[43] Larsson SC, Orsini N and Wolk A. Body mass index and pancreatic cancer risk: A meta-analysis of prospective studies. International Journal of Cancer. 2007; 120(9): 1993–1998. DOI: 10.1002/ijc.22535 17266034

[44] Smith JP, Strauss J and Zhao Y. Healthy aging in China. Journal of the Economics of Aging. 2014; 4: 37–43. DOI: 10.1016/j.jeoa.2014.08.006 PMC4303107 25621202

[45] Kahn BB and Flier JS. Obesity and insulin resistance. The Journal of Clinical Investigation. 2000; 106(4): 473–481. DOI: 10.1172/JCI10842 10953022 PMC380258

[46] Van Kruijsdijk RC, Van Der Wall E and Visseren FL. Obesity and cancer: the role of dysfunctional adipose tissue. Cancer Epidemiology and Prevention Biomarkers. 2009; 18(10): 2569–2578. DOI: 10.1158/1055-9965.EPI-09-0372 19755644

[47] Reulen RC, de Vogel S, Zhong W, et al. Physical activity and risk of prostate and bladder cancer in China: The South and East China case-control study on prostate and bladder cancer. PloS One. 2017; 12(6): e0178613 DOI: 10.1371/journal.pone.0178613 28575110 PMC5456085

[48] Cannioto R, Etter JL, Guterman LB, et al. The association of lifetime physical inactivity with bladder and renal cancer risk: A hospital-based case-control analysis. Cancer Epidemiology. 2017; 49: 24–29. DOI: 10.1016/j.canep.2017.04.017 28528291 PMC5544555

[49] Moore SC, Lee I-M, Weiderpass E, et al. Association of leisure-time physical activity with risk of 26 types of cancer in 1.44 million adults. JAMA Internal Medicine. 2016; 176(6): 816–825. DOI: 10.1001/jamainternmed.2016.1548 27183032 PMC5812009

[50] Kyu HH, Bachman VF, Alexander LT, et al. Physical activity and risk of breast cancer, colon cancer, diabetes, ischemic heart disease, and ischemic stroke events: Systematic review and dose-response meta-analysis for the Global Burden of Disease Study. BMJ. 2013; 354: i3857 DOI: 10.1136/bmj.i3857 PMC4979358 27510511

[51] Jee SH, Samet JM, Ohrr H, Kim JH and Kim IS. Smoking and cancer risk in Korean men and women. Cancer Causes & Control. 2004; 15(4): 341–348. DOI: 10.1023/B:CACO.0000027481.48153.97 15141135

[52] Parkin DM. The global burden of urinary bladder cancer. Scandinavian Journal of Urology and Nephrology. 2008; 42(sup 218): 12–20. DOI: 10.1080/03008880802285032 19054893

[53] Institute for Health Metrics and Evaluation. GBD Cause Patterns Seattle, WA: Institute for Health Metrics and Evaluation, University of Washington; 2013 vizhub.healthdata.org/gbd-cause-patterns/. Accessed September 15, 2014.

[54] Vrieling A, Bueno-de-Mesquita HB, Boshuizen HC, et al. Cigarette smoking, environmental tobacco smoke exposure and pancreatic cancer risk in the European Prospective Investigation into Cancer and Nutrition. International Journal of Cancer. 2010; 126(10): 2394–2403. DOI: 10.1002/ijc.24907 19790196

[55] Ezzati M, Henley SJ, Lopez AD and Thun MJ. Role of smoking in global and regional cancer epidemiology: Current patterns and data needs. International Journal of Cancer. 2005; 116(6): 963–971. DOI: 10.1002/ijc.21100 15880414

[56] Krejs GJ. Gastric cancer: Epidemiology and risk factors. Digestive Diseases. 2010; 28(4–5): 600–603. DOI: 10.1159/000320277 21088409

[57] Gaudet MM, Gapstur SM, Sun J, Diver WR, Hannan LM and Thun MJ. Active smoking and breast cancer risk: Original cohort data and meta-analysis. Journal of the National Cancer Institute. 2013; 105(8): 515–525. DOI: 10.1093/jnci/djt023 23449445

[58] Xue F, Willett WC, Rosner BA, Hankinson SE and Michels KB. Cigarette smoking and the incidence of breast cancer. Archives of Internal Medicine. 2011; 171(2): 125–133. DOI: 10.1001/archinternmed.2010.503 21263102 PMC3131146

[59] Catsburg C, Miller AB and Rohan TE. Active cigarette smoking and risk of breast cancer. International Journal of Cancer. 2015; 136(9): 2204–2209. DOI: 10.1002/ijc.29266 25307527

[60] Cheng J, Chen Y, Wang X, et al. Meta-analysis of prospective cohort studies of cigarette smoking and the incidence of colon and rectal cancers. European Journal of Cancer Prevention. 2015; 24(1): 6–15. DOI: 10.1097/CEJ.0000000000000011 24722538

